# Technical Implications of the Chicken Embryo Chorioallantoic Membrane Assay to Elucidate Neuroblastoma Biology

**DOI:** 10.3390/ijms241914744

**Published:** 2023-09-29

**Authors:** Carlos César Patiño-Morales, Ricardo Jaime-Cruz, Tania Cristina Ramírez-Fuentes, Laura Villavicencio-Guzmán, Marcela Salazar-García

**Affiliations:** 1 Developmental Biology Research Laboratory, Hospital Infantil de México Federico Gómez, Mexico City 06720, Mexico; cpatino@cua.uam.mx (C.C.P.-M.); ricardo.jaime.cruz@gmail.com (R.J.-C.); tania.ramirez3190@outlook.com (T.C.R.-F.); villagu@yahoo.com (L.V.-G.); 2 Cell Biology Laboratory, Universidad Autónoma Metropolitana-Cuajimalpa, Mexico City 05348, Mexico; 3 Department of Health Sciences, Universidad Tecnológica de México-UNITEC México-Campus Sur, Mexico City 09810, Mexico; 4 Section of Graduate Studies and Research, School of Medicine of the National Polytechnic Institute, Mexico City 11340, Mexico; 5 Facultad de Medicina, Universidad Nacional Autónoma de México, Mexico City 04360, Mexico

**Keywords:** neuroblastoma, CAM, tumors, cancer

## Abstract

The chorioallantoic membrane (CAM) can be used as a valuable research tool to examine tumors. The CAM can be used to investigate processes such as migration, invasion, and angiogenesis and to assess novel antitumor drugs. The CAM can be used to establish tumors in a straightforward, rapid, and cost-effective manner via xenotransplantation of cells or tumor tissues with reproducible results; furthermore, the use of the CAM adheres to the three “R” principle, i.e., replace, reduce, and refine. To achieve successful tumor establishment and survival, several technical aspects should be taken into consideration. The complexity and heterogeneity of diseases including neuroblastoma and cancers in general and their impact on human health highlight the importance of preclinical models that help us describe tumor-specific biological processes. These models will not only help in understanding tumor biology, but also allow clinicians to explore therapeutic alternatives that will improve current treatment strategies. In this review, we summarize the technical characteristics as well as the main findings regarding the use of this model to study neuroblastoma for angiogenesis, metastasis, drug sensitivity, and drug resistance.

## 1. Introduction

The chorioallantoic membrane (CAM) is a vital extra-embryonic structure that significantly influences embryonic development and encompasses diverse physiological functions. Furthermore, the CAM is highly vascularized and lacks innervation; thus, it has been used to test natural products or drugs with potential antitumor activity. Owing to these features, the CAM is considered an ideal candidate to help in the establishment of tumors. This consequently enables the investigation of cancer developmental biology, which includes processes of tumor growth, angiogenesis, and metastasis [1,2].

## 2. Structure and Origin of the CAM

During chick embryo development, the allantois emerges as a caudal evagination of the yolk sac at approximately 3.5 days of incubation. The allantoic vesicle subsequently undergoes rapid expansion between days 4 and 10 of incubation, which leads to CAM formation through the fusion of the mesodermal layer of the allantois with the adjacent mesodermal layer of the chorion. The CAM is a highly vascularized double-membrane structure that is connected to the general circulation of the embryo by two allantoic arteries and one allantoic vein. The CAM is fully differentiated and functional by days 12 and 19, respectively [3,4].

The CAM has several important roles during development, i.e., it has supportive and excretory functions, serves as a respiratory organ, participates in osteogenesis, and transports sodium and chloride [5].

Proteomic analysis of the CAM on days 12 and 19 has unveiled an extensive array of proteins related to its physiology. These proteins include those involved in calcium ion transport (ATPases, CaBP, and carbonic anhydrases); gas exchange (annexin, hemoglobin, and tubulin); vascularization processes (collagen, integrins, and laminins); and protective molecules against pathogens (cystatins and serpins) [3]. In terms of angiogenesis, the process is predominantly regulated by growth factors with diverse activities including regulatory, proliferative, and inhibitory functions. Notably, in the context of CAM, the following regulatory factors have been reportedly involved in this process: VEGF (vascular endothelial growth factor), FGF (fibroblast growth factor), PDGF (platelet-derived growth factor), ANG (angiopoietin), HGF (hepatocyte growth factor), HIF (hypoxia-inducible factor), and endostatin. The chick embryo CAM is a structure commonly used to study blood vessel development and to evaluate molecules with pro- or anti-angiogenic properties [1,3].

The CAM model was initially utilized in 1911 for the growth of chicken sarcoma [5,6]. Tumors can be induced through xenotransplantation of tumor cell lines, human tumor grafts, or even mouse tumor xenografts. This model has garnered significant interest due to its ability to replicate certain microenvironmental conditions, facilitating a deeper understanding of tumor biology. Several types of tumors have been successfully implanted in the chick embryo CAM [7], including colon tumors [8,9], melanoma [10,11], glioblastoma [12,13], sarcoma [14,15], ovarian [16,17], renal cell carcinoma [18,19], urothelial carcinoma [20], prostate cancer [21] nasopharyngeal and head and neck cancer [22,23], laryngeal squamous cell carcinoma [24], giant cell tumor of bone [25], lymphoma [26], hepatocellular carcinoma [27,28], and pancreatic cancer [29,30]. These results are achieved within a short period of time, and when compared with other tumor induction models, the CAM is a very low-cost model.

## 3. Technical Characteristics of the Use of CAM for In Ovo Tumor Generation

**Figure 1:**
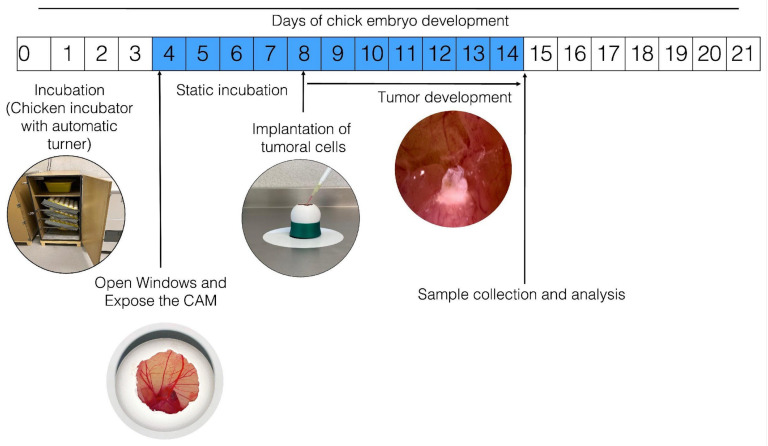
General procedure for in ovo tumor induction in chorioallantoic membrane (CAM). The numbers inside the boxes represent the days of incubation that the chick embryo requires to develop until hatching; the blue color indicates the days from the formation of the CAM until it is fully functional. Fertilized hen eggs in a dynamic incubator: on day 4, open window in eggshell and expose the CAM; tumor cells are inoculated on day 8; the inoculated eggs are placed in an incubator without movement for the time required for the experiment; the tumor is observable after three days of incubation.

Figure 1 schematizes how the process of establishment of tumors occurs in the CAM of the chicken embryo; however, variations or adaptations may respectively occur or be implemented as per the interests of the clinician. To develop this technique, fertilized chicken eggs are placed in an incubator, preferably with automatic rotation, at 85% relative humidity and 37.5 °C. On the fourth day of incubation (when the CAM has formed), the eggs are removed from the incubator and disinfected using gauze sprayed with 70% alcohol (ethanol should not be sprayed directly on the egg to avoid affecting embryo survival). Small sections of the eggshell are then gently removed from the blunt end of the egg using dissecting tweezers. This is done to create a window (~1 cm^2^) in the eggshell to expose the external testaceus membrane. This membrane is gently blown out using a bulbous Pasteur pipette (to prevent small shell fragments from falling into the air chamber) and removed. This exposes the inner testaceus membrane, which forms the bottom of the air chamber; this membrane must be removed very carefully so as not to damage the vasculature, thus exposing the CAM for tumor cell inoculation. Finally, the window is sealed using transparent tape and the egg is placed in a static incubator at 37.5 °C with constant humidity (85%). Typically, the induced tumor becomes observable 2–3 days after inoculation. Some authors have stated that trauma or a laceration should be performed on the CAM before inoculating the cells because this facilitates the installation of the tumor. From a cellular perspective, in order to settle in the CAM, the cells must be able to produce MMPs; although many neuroblastoma-derived cells secrete them, only SKNAS cells are capable of also expressing the activator. SKNAS cells form tumors very efficiently, but for other cell types such as IMR32, Kelly, and BE2C, trypsin is required to enhance tumor formation [31]. It has also been reported that in the event that the inoculated cells are highly metastatic, it is not necessary to lacerate the CAM [32].

## 4. In Ovo and Ex Ovo CAM Assay

Working with an embryo inside an egg (in ovo model) offers several advantages, such as preserving the physiological environment for oviparous development and ensuring a continuous calcium source; furthermore, it is safer for the normal development of the embryo. This model exhibits high survival rates for manipulated embryos and does not demand a completely sterile environment for its manipulation. In addition, this model has the following disadvantages: it limits the working area and makes it difficult to observe what has been implanted in the CAM, and when the shells are opened, pieces of the shell (angiogenesis inducers) can fall into the egg and interfere with angiogenesis studies. Alternatively, the ex ovo model may be used, in which the embryo together with its extra-embryonic membranes can be transferred to a petri dish (usually a 6-well or 35 mm dish) on day 3 or 4 of incubation. In this modality the CAM develops as a monolayer, thereby allowing the placement of multiple implants. Embryo accessibility is enhanced, and researchers have a relatively larger area to make observations or place grafts; however, the long-term viability is usually lower in the ex ovo model than in the in ovo model. Ensuring that the embryos remain hydrated during the experiments is of paramount importance [33]. The ex ovo model presents a higher risk of contamination, and this point must be taken into consideration when working with this model. Additionally, the ability to open the egg and transfer the embryo to the petri dish without injuring it, without rupturing or mistreating the extra-embryonic membranes, and without disrupting the vasculature must be considered.

## 5. CAM Assay as an Alternative to the Use of Experimental Animals

Ethical codes including the Nuremberg Code and the Declaration of Helsinki emphasize that biomedical research involving human subjects should be based on properly conducted laboratory tests and animal experiments. When animals are used in research, researchers have a legal and moral obligation to ensure the animals’ welfare and to minimize their suffering. The use of the CAM assay to study the developmental biology of NB is one of the ethical ways to replace, reduce, and minimize use of animals in research, thereby complying with the principle of the three “R’s.”

Tests performed on the CAM do not involve the use of complex procedures, consequently waiving the need to obtain approval for use. Considering that the CAM is not innervated, experiments can be performed before the embryo perceives pain. The Institutional Animal Care and Use Committee (IACUC), the Association of New England Medical Center and Tufts (IACUC, 2001), and the National Institute of Health, USA (National Institute of Health, 1991) state that a chick embryo does not experience pain before day 14 of development; thus, before that day, it can be used for experimentation [34,35].

Compared with mammalian models where tumor growth can be slow and require up to six weeks, induction of tumors in the chick embryo CAM is much faster. Furthermore, tumor induction is typically successful within approximately 2–5 days. This rapid tumor development in the CAM makes it an excellent experimental strategy.

Since the CAM is a highly vascularized environment, vascularization can be observed in the tumors that subsequently develop. Tumor cells can either remain within the CAM or invade the organs of the embryo and spread to the lungs, liver, and brain. This has made this model useful for both angiogenesis and metastasis studies [30]. Additionally, the naturally immunodeficient chicken embryo can receive transplants of different tissues and species without the risk of rejection due to immune response [33,36]. There are also limitations of the model; for example, the number of reagents compatible with avian species, including antibodies, cytokines, and primers, and genetic manipulation can be more complicated than in mice; sometimes it is difficult to discriminate between new and existing capillaries; experiments cannot be prolonged for such a long time; a non-specific inflammatory reaction can occur that can limit the success of the xenograft; and it is not possible to examine tumor–immune cell interaction. Another important point is that model is also extremely sensitive to modification by environmental factors, such as changes in oxygen tension, pH, temperature, and osmolarity, risk of angiogenesis induced by technical errors (eggshell pieces), differences in drug metabolism and immune system with mammals; furthermore, the oral route cannot be tested [35,36,37]. Table 1 compares the CAM model with the mouse model and cell cultures.

Indeed, the chick embryo CAM model exhibits a multitude of behavioral, anatomical, biochemical, pharmacological, and angiogenic similarities with the abovementioned model, making it a valuable alternative for investigating potential therapeutic approaches in cancer treatment. The preclinical data generated using this non-sentient animal model are indispensable to understand the developmental biology of tumors and the underlying molecular mechanisms. Furthermore, these data aid in determining whether the benefits of a treatment outweigh the risks of adverse side effects and in establishing safe dosages for use in human clinical trials.

Although the focus of this review is on the use of the CAM to study tumor biology, it is essential to recognize that the applications of the CAM are not limited solely to this area. The CAM model can also be used for the study of non-tumor tissue grafts, testing the biocompatibility of materials used in tissue engineering, for wound healing studies, toxicology and drug delivery studies, and the study of molecules with angiogenic or anti-angiogenic effects, among others [35]. The aim of this review is to summarize the main technical characteristics of using the CAM to induce NB tumors and to present the main results obtained through the use of this model in the different studies performed thus far. Therefore, for those researchers who wish to use this model to study NB or even other tumors, we intend this work to serve as a basis on which to establish the model.

## 6. The Importance of Studying NB

NB is a malignant tumor that arises from neural crest cells and can develop anywhere in the sympathetic nervous system, and is commonly found in the adrenal medulla and paravertebral ganglia. It is one of the most common extracranial solid tumors in children. Most of the cases of NB are diagnosed before the age of five years and a median age of diagnosis of 17 months. NB displays significant heterogeneity, which is evident in varying survival rates among patients. Based on clinical and molecular factors, patients with NB can be categorized into very low risk, low risk, intermediate risk, high risk, or very high risk [49,50]. Low- to intermediate-risk patients have been reported to have a survival rate of more than 95%, whereas high-risk patients have a long-term survival rate of less than 50% [51]. NB rarely occurs in adolescents and young adults; however, upon its occurrence it generally has a poor prognosis. The International Neuroblastoma Risk Group Staging System (INRGSS) provides a classification to uniformly define the risk of patients with NB according to genomic and molecular biomarkers, including chromosomal aberrations, mutational profiles, and telomere maintenance mechanisms. The classification is based on two stages of localization (L1 and L2) and two stages of metastasis (M and MS). The stage is considered L1 if the tumor has no image-defined risk factors (IDRFs); L2 if the tumor has one or more IDRFs; M if distant metastases are present (except MS); and MS in an L1 or L2 tumor with the presence of skin, liver, and/or bone marrow metastases. By definition, high-risk NB is a metastatic tumor in a child aged ≥18 months or of any age with L2, M, or MS disease with MYCN oncogene amplification [52]. Although NB is characterized by the presence of chromosomal aberrations including the deletion of a distal part of the short arm of chromosome 1, gain of the long arm of chromosome 17, and loss of part of the long arm of chromosome 11 [53,54], more than 50% of patients with NB can develop metastases in organs including lymph nodes, bone marrow, bone, liver, and skin. Some patients present with specific clinical signs and symptoms, including Horner’s syndrome and spinal cord compression, caused by local damage or infiltration of primary lesions into the nervous system [55]. NB treatment has advanced significantly in recent years and now includes immunotherapy in addition to conventional chemotherapy, radiotherapy, and surgery [56,57]. However, approximately half of high-risk NB patients do not respond to the first-line treatment protocol or relapse within the first 2 years after treatment [58]. This highlights the need for ongoing research into NB and the implementation of preclinical models that will allow rapid progress in the development of new therapies that are more effective, but at the same time do not compromise the quality of life of patients.

From a therapeutic standpoint, it is now known that the tumor microenvironment plays a critical role in establishing a successful therapy. In the context of NB, innate and adaptive immune cells have received much attention, but non-immune cells are also important (endothelial cells, cancer-associated fibroblasts, pericytes, and mesenchymal cells). Furthermore, other cellular aspects, such as alterations in the extracellular matrix (ECM), the presence of extracellular vasculature, and the presence of extracellular vesicles released by cells in the tumor microenvironment (carrying oncogenic microRNAs and proteins involved in tumor progression such as CD147, CD276/B7-H3), play a key role in both tumor progression and the development of resistance to therapy [59]. Although in vitro NB studies have been very useful in describing some features of the cellular and molecular biology of these tumors, the establishment of in vivo models will allow a better study of tumor characteristics in relation to the microenvironment.

## 7. CAM and NB

In the investigation of human NB, it is noteworthy that, besides the chicken model, the quail CAM model has also been used. For example, Azar and collaborators developed an ex ovo CAM assay to study IGFBP-2 mediated angiogenesis. The methodology involved incubating fertilized quail eggs for 48 h, transferring the contents to a six-well plate, and inoculating cells into collagen sponges for vascularization analysis for up to 72 h [60,61]. Similarly, Hecht’s group used the quail in ovo model to describe how the neurotrophin receptor TrkB cooperates with c-Met to promote NB invasiveness. For this purpose, 3 × 10^6^ cells were inoculated in 50 μL of medium in a ring (Thermanox) in the CAM of an 8-day incubated embryo. Six days after inoculation, the resulting tumors were analyzed [62].

Ribatti and coworkers pioneered the establishment of the chicken CAM model for studying NB [63]. Although to date this model has not been used for the study of neuroblastoma-derived cancer stem cells, a recent paper reported that the chicken CAM model can be highly useful for inducing tumors from circulating cancer stem cells in patients with breast cancer. In cancer research, the study of cancer stem cell subpopulations is extremely important as they are responsible for patient relapse and metastasis. The authors describe that a culture of tumospheres enriched in cancer stem cells was generated from circulating cells. These tumospheres were placed in the CAM to generate tumors; five of the ten patient samples used induced tumors. Morphologically, these tumors are vascularized and capable of invading the CAM. Histologically, the tumors closely resembled primary tumors observed in the patients. Therefore, this study reported that circulating cancer stem cells from patients with cancer (tumospheres positive for EpCAM and CD 44 +, and CD24− or CD24 low and positive ALDH1 activity) can be implanted in the CAM to generate tumors. As a preclinical model, the CAM may have important applications in not only studying the biology of this cell subpopulation, but also for evaluating new therapeutic alternatives aimed at eliminating these cells [64].

Current research utilizing CAM models has focused on optimizing tumor analysis techniques. For instance, Eckrich et al. used repetitive ultrasonography to monitor the growth and vascularization of tumors induced in the CAM using the HuH7 cell line (hepatic carcinoma). This innovative methodology has proven to be particularly useful for quantifying tumor size and vascularization, one of the advantages of which is that tumor development can be continuously monitored in the in ovo model [65]. Therefore, the efficiency of analyzing the samples generated in this model can be improved when complemented with current technological advances. Repetitive ultrasonography could also be used for the analysis and follow-up of NB tumors generated in the CAM.

**Figure 2:**
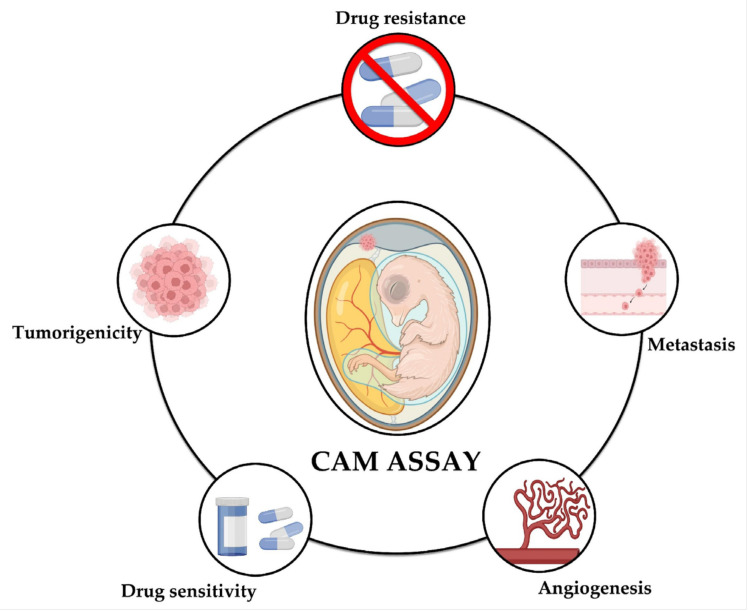
The CAM model for the study of neuroblastoma. The CAM assay has shown to be a useful tool for the study of different aspects of neuroblastoma, namely tumorigenicity, metastasis, angiogenesis, drug sensitivity, and drug resistance.

The following presents the main findings reported through the use of the CAM assay in different studies on NB that have been reported to date; the technical characteristics of the methodology are summarized in Table 2 and Figure 2 shows the aspects of neuroblastoma that can be studied using the CAM model: tumorigenicity, angiogenesis, metastasis, drug sensitivity, and drug resistance.

## 8. Using CAM Assay to Study Metastasis and Antimetastatic Drugs

Due to its accessibility, high vascularity, ease of use, and natural immunodeficiency, the CAM can serve as a valuable model for studying tumor metastasis induced from cell lines, from patient samples, or via inoculation of tumor tissue from previously induced tumors in mice. For non-solid tumors such as leukemia, tumor cells can be injected directly into the allantoic vein. Metastasis analysis using the CAM model can be conducted in different ways; on the one hand, it is possible to detect and identify tumor cells that have migrated into the blood vessels from a primary tumor, or to locate cells that invade the organs of the embryo. On the other hand, the model also allows the analysis of the tropism of different cell lines or tumor cells. Techniques for metastasis analysis from tumors induced in the CAM range from classical histological techniques, including staining of tumor tissue sections with H&E or immunohistochemistry and immunofluorescence (where cells can be seen migrating into blood vessels), to molecular techniques, such as amplification of Alu sequences (primordial sequences) via polymerase chain reaction (from samples of genetic material obtained from different organs of the embryo after the tumor has been induced in the CAM) [66]. Metastasis is the primary cause of death in patients with cancer, and the CAM assay has greatly aided studies examining this underlying process. Despite the presence of in vitro models capable of measuring the migratory or invasive process in cells, in vivo models are necessary. For instance, Merlos Rodrigo et al., in 2021, used both the in ovo and ex ovo CAM assays to determine the antimetastatic effect of cisplatin (CDD) and ellipticin (Elli) in tumors induced from the UKF-NB-4 NB line. The ex ovo model was used to determine whether CDD and Elli were able to reduce tumor size and inhibit tumor cell extravasation. The in ovo model was used to learn whether these drugs could inhibit metastasis to different organs. The survival rate was over 50% in the ex ovo model and 100% in the in ovo model after tumor induction. Regarding the treatment, doses of 100 μM CDDP (cis-diamminedichloroplatinum) or 200 μM Elli were used, which were added over each microtumor generated in the CAM for 24 h. The authors observed that both drugs reduced the size of the tumors, decreased the extravasation of tumor cells into the blood vessels distal to the inoculation site, and decreased the number of cells that spread to other organs in the chick, including liver, lung, and brain. The authors conclude that both the in ovo and ex ovo models are very useful for studying drugs that may have applicability by inhibiting tumor proliferation and metastasis [67].

In another study, researchers used the chicken CAM model to evaluate how hypoxic preconditioning of cells promotes metastasis. NB cells stably transfected with fluorescent proteins, specifically SK-N-AS cells expressing EGFP and dTomato, were implanted into the chicken CAM on day 7. The following two groups of cells were pre-cultured: SK-N-AS-dTomato cells were exposed to 21% O_2_ for 3 days, whereas SK-N-AS-EGFP cells were exposed to 1% O_2_ for 3 days. Both sets of cells were co-inoculated to assess whether invasion of normoxic cells can be initiated via direct interaction with hypoxic cells. The results showed that metastasis occurred not only in cells pre-cultured under hypoxia, but also in cells cultured under normoxic conditions. In addition, tumors were induced with another set of cells that were chemically hypoxic by treating them with 0.5 mM DMOG (dimethyloxaloylglycine); these cells are capable of producing microtumors; hypoxic preconditioning should consist of at least 3 days at 1% O_2_ to promote extravasation and metastasis. The labeling of cells with fluorescent proteins facilitated cell tracking once they were implanted in the CAM—a particularly useful strategy when migration and metastasis studies are required. The results showed that exposure of NB cells to 1% oxygen for 3 days had several important effects, as it promoted cell migration into blood vessels, facilitated extravasation, and favored cell proliferation in primary and secondary sites [68,69].

Similarly, the research group of Al-Mutawa et al., in 2018, performed a study on metastasis and hypoxia using the chicken CAM model. They implanted fluorescently labeled NB cells preconditioned in either 1% or 21% O_2_ for 3 days. The inoculation was performed on day 7, and on day 14, intratumoral oxygen tension was measured. Owing to the presence of a relationship between tumor tissue oxygen tension and cellular metabolic activity, this working group hypothesized that metabolic profiling between metastatic and non-metastatic tumors would reveal new potential biomarkers. The researchers conclude that NB tumors formed in the chick embryo CAM have a heterogeneous oxygenation profile, with a predominantly hypoxic environment (<1.5% O_2_). Moreover, these tumors exhibited specific metabolite profiles including decreased carbohydrate, ATP, and taurine and increased ADP, isoleucine, and glutamate [70].

Herrmann et al. performed a study using magnetic resonance imaging (MRI) technology for the characterization of a model of NB-derived cell metastasis in chick embryos. To induce tumors, the researchers inoculated 1 × 10^6^ SK-N-AS and SK-N-AS-labeled cells resuspended in 2 microliters of serum-free medium at 7 days of incubation. For observation of spontaneous metastasis, implantation was performed in the CAM at day 7. Prior to implantation, SK-N-AS cells were preconditioned in 1% O_2_ for 3 days. Tumor formation was monitored using MRI, which proved to be a suitable and highly sensitive technique for the studying tumorigenesis in ovo. It also allows the detection of metastatic deposits in the embryo. The authors determined that the lower limit of detection for tumor cells using MRI was 12 cells. The MRI used in this model allows a longitudinal view of tumor progression in the same animal in a non-invasive manner. The cells were labeled with micron-sized iron particles (MPIOs) to facilitate their visualization via MRI when the tumor is established. Furthermore, although labeling does not have significant advantages for the detection of primary tumors compared with unlabeled cells, it is necessary for the detection of small metastases in chick embryo organs [71].

An in vivo analysis was performed at the University of Santiago de Chile, using the chick embryo CAM model, to determine whether NEO1 and NTN4 are involved in the extravasation and metastasis of tumors induced by NB cell lines. They used the SK-N-SH cell line, of which ten million cells were inoculated and divided into the following groups: shNEO1 silencing (NEO1), shNTN4 (NTN4 silencing), and shSCR (a control or scramble). After using the CAM assay, the authors reported that NTN4 and its receptor NEO1 promote cell migration, survival, and metastasis in NB-derived cells. Specifically, in the absence of NTN4 expression, NEO1 induces apoptosis, whereas in the presence of NTN4, NEO1 signaling promotes cell survival and migration [72]. The CAM assay has been shown to be a good model for the study of metastasis; tumors induced from cell lines derived from neuroblastoma can metastasize to the liver, lung, and brain [67], but in other cases, tumor entities have also been reported to be able to induce metastasis in the eye, heart, and kidneys [73], due to the advantages of the model, such as access to the vasculature, metastasis that occurs spontaneously from tumors generated in the CAM, and direct injection of the cells, which can also be performed through the chorioallantoic vein. The cells are capable of crossing the vessels and have a high survival in circulation (±80%); in addition, it has been reported that metastasis can be visualized from the presence of secondary tumors due to cells that have migrated from the primary transplant site (CAM) [74]. Labeling the cells that are inoculated with fluorescent molecules allows them to be monitored; immunohistochemistry techniques have also made it possible to observe tumor cells invading chicken tissues [75]. Because there is a difference between the species from which the tumor originates (mouse or human) and the recipient (chicken) in the model, the amplification of mammalian genes (PCR or qPCR) from the genetic material obtained in the organs of chicken after tumors have been induced in the CAM is evidence of metastatic processes [76,77,78].

## 9. Using the CAM Assay to Study Chemoresistance and Molecules with Antitumor Effect in NB

In the study performed by Rodrigo et al., 2021, they used the CAM assay to investigate the impact of human metallothionein 3 (hMT3) overexpression in NB-derived cells and its role in cisplatin resistance. They used NB-derived cells (UKF-NB-4) and cisplatin-resistant cells (UKF-NB-4CDDP) for tumor induction. The result indicated 100% embryo survival in the inoculated eggs. UKF-NB-4 xenografts treated with CDDP (100 μM) showed growth inhibition (approximately two-fold) of tumor weight. Furthermore, the number of cells in the liver, lung, and brain tissues was significantly reduced compared to that in controls. Notably, CDDP did not exhibit a significant inhibitory effect on tumor growth (weight) or metastasis in UKF-NB-4 cells, highlighting the chemoresistance of these cells to CDDP and its inability to inhibit metastasis. The authors confirmed that overexpression of hMT3 resulted in the development of CDDP chemoresistance. Tumor weights in control and hMT3-expressing tumor cells were similar (91.6 mg vs. 86.7 mg, respectively). Using the CAM assay, the authors demonstrated for the first time that hMT3 promotes tumor cell intravasation and organ metastasis [79].

Swadi et al., 2019, describe the use of the CAM to test the antitumor efficacy of two kinase inhibitor compounds; they used SK-N-AS and BE(2)C cell lines to induce tumors. Notably, it is interesting to mention that, to optimize tumor generation, these authors reported that it was necessary to injure the CAM with a pipette tip or traumatization using a strip of sterile lens tissue to produce a small bleed followed by the addition of 5 µL of 0.05% trypsin 0.5 mM EDTA; other authors had already reported this procedure. The authors observed that CDK4/6 (palbociclib) and CDK1 (RO-3306) inhibitors led to a reduction in cell proliferation in tumors induced with BE(2)C and SK-N-AS lines (by Ki67 marker analysis); treatment was administered on days 11 and 13 after tumor induction). In addition to inhibiting proliferation, RO-3306 reduced metastasis by nearly 60%, whereas palbociclib reduced metastasis by 56% [80]. In another publication by the same research group, the CAM assay was used to induce NB-derived tumors from IMR32 and SK-N-BE(2)C lines, and the rupture of the chorialantoid membrane and the use of trypsin were performed under the same conditions as described at the beginning of the paragraph. In this work, 2 × 10^6^ cells were also seeded to induce tumors in the CAM; once induced, the tumors were treated with ATRA (all-trans retinoic acid). Treatment with ATRA at a concentration of 100 μM on days 11, 12, and 13 induced a change in the expression of differentiation markers (upregulation of STMN4 and ROBO2 and downregulation of KLF4) and led to a 43% reduction in cell proliferation. However, treatment with ATRA at a concentration of 40 μM on days 11 and 13 reduced cell proliferation by 37%, which was accompanied by a change in cell morphology within the tumor [81].

The continued search for drugs with antitumor activity has revealed that the antifungal benzimidazoles, including mebendazole and flubendazole, exhibit antitumor activity through mechanisms that include inhibition of microtubule function. Michaelis et al. tested the effect of flubendazole treatment on the viability of 461 tumor cell lines, among which NB showed high sensitivity to flubendazole. To analyze the effects of flubendazole on the growth of NB tumors in the chicken CAM, three cell lines were used: the UKF-NB-3 line and its cisplatin-resistant subline UKF-NB-3r CDDP1000, and UKF-NB-3 subline VCRr10, which is resistant to vincristine. Cells were isolated from bone marrow aspirates of five metastatic patients (with amplified MYCN) classified with INSS stage 4 NB. Five million cells were inoculated into the chicken CAM on day 11 of incubation and allowed to grow for 3 days. Treatment with 2 μg flubendazole resulted in reduced tumor growth and the presence of large necrotic areas. In addition, the authors demonstrated that the treatment reduced cell invasion on the inner side of the CAM [82].

The induction of neuroblastoma tumors in the CAM has proven to be an efficient model for the evaluation of new antitumor drugs or for chemoresistance to already-known antitumor drugs, due to the easy access to the tumor and the vasculature of the embryo. As a result, a larger number can be used in all tests, to experiment with a large number of medications and doses, and thus test different routes of administration, since the medications can be introduced into the embryo and extraembryonic tumors by topical addition, intravenous injection, or injection into the allantois [37,81,83]; even the intraperitoneal route of administration has been reported [84]. The model has even allowed the exploration of routes of drug administration that do not have an equivalent in humans such as the yolk sack and the amnion [85,86].

## 10. Studying Angiogenesis in NB Using the CAM Assay

Studies of angiogenesis in NB are important as tumor progression is closely linked to a high vascular index. In a 1998 study, tumors were induced in the CAM using LAN-5 and GI-LI-N cell lines (from human NB). This research not only demonstrated the ability of NB cells to produce ECM-degrading enzymes in vitro (MMP-2 and MMP-9), but also demonstrated their angiogenesis capacity in vivo. The experimental design involved implantation of sponges containing a suspension of LAN-5 or GI-LI-N cells in chicken CAM (after 8 days of incubation). Negative controls used sponges with RPMI-1640 alone, while positive controls included VEGF and FGF2. After 12 days of incubation, it was macroscopically observed that the sponges with cells and those used as a positive control were surrounded by radial blood vessels, demonstrating the angiogenic capacity of human NB cell lines, and also demonstrating that the model can be very useful in the study of the biology of the human NB tumor [87]. In NB, more aggressive tumors often exhibit MYCN gene amplification. Therefore, Ribatti’s group used the CAM assay to study how MYCN amplification relates to the angiogenic potential of NB-derived cells. For this purpose, they placed two types of samples in the CAM; 1–2 mm^3^ fragments from biopsies of NB patients and 1–2 mm^3^ fragments from biopsies obtained from xenografts derived from four cell lines (HTLA-230; GI-LI-N (MYCN-amplified); ACN; SH-SY5Y (MYCN-nonamplified)) injected into nude mice. The results demonstrated that the angiogenic response, assessed both macroscopically and microscopically, was significantly higher in MYCN-amplified samples than in those without amplification [88].

Furthermore, the impact of FOXO3 on angiogenesis in NB was investigated. The working group of Hagenbuchner and coworkers used the chicken model for tumor induction. They inoculated 2 × 10^6^ cells of STA-NB15 (a cell line derived from patient tumors) and another group of cells with ectopic expression of FOXO3 (NB15/FOXO3) into the chicken CAM on day 10. The authors concluded that FOXO3 promotes tumor growth under hypoxic conditions and promotes tumor angiogenesis in advanced-stage NB tumors [89].

In the context of studying angiogenesis in NB, Klingenber’s research group used the CAM assay to investigate the potential therapeutic targeting of angiopoietin-2 (Ang-2) in human NBs. They used cells in which Ang-2 expression was negatively regulated using an shRNA. Three types of cells were separately inoculated in the chicken CAM, i.e., Kelly, scrambled Kelly (shRNA control), and Ang-2 Kelly (shRNA against Ang-2). The results showed that tumor growth was reduced in chicken CAM when Ang-2 was negatively regulated, and the density of microvessels present in tumors derived from Kelly cells with downregulated Ang-2 (cells transduced with shRNA against angiopoietin-2) was also decreased [90].

Martin Michaelis and coworkers established a standardized model of NB tumorigenesis using chemoresistant cell lines. Through bioinformatics analysis, they first observed that the expression of angiogenesis-associated genes showed differences between chemosensitive and chemoresistant NB cells. To perform the angiogenesis analysis, the authors used the chicken CAM assay, using the UKF-NB-3 cell line isolated from patients with stage 4 NB (N-myc amplified) and bone marrow metastases. These cells were engineered to grow in the presence of drugs as follows: UKF-NB-3 VCRr10 adapted to grow in the presence of vincristine (10 ng/mL); UKF-NB-3r DOX20 adapted to grow in doxorubicin (20 ng/mL); and UKF-NB-3r CDDP1000 adapted to grow in the presence of cisplatin (1000 ng/mL). To assess the impact of the chemoresistance of these cells on tumor angiogenesis, the chicken CAM (together with non-chemoresistant cells) was inoculated with 10^6^ cells in 50 μL of medium on day 8 of incubation. Vascularization was assessed on day 12. Doxorubicin treatment of doxorubicin-resistant NB xenografts resulted in a decrease in blood vessel formation; however, a concurrent increase in tumor size was observed, suggesting that chemoresistance may contribute to the increased malignancy of NB cells [91].

To assess the angiogenic potential of NB-derived cells, Mangieri et al. used the CAM assay. They inoculated the NB-derived cell line IMR-32, using silicon rings with 2 μL of NB cell suspension to induce tumors, and compared it with RPMI-1640 medium alone or supplemented with FGF-2 (200 μg/mL). Through this model, the researchers observed that NB cells induce an angiogenic response comparable to that induced by FGF-2. The tumor exhibited high expression of human VEGF-A, FGF-2, ANG-1, and HIF-2a, and a low expression of a human endostatin. Therefore, the growth of NB cells in chicken CAM shows characteristics similar to those found in human tumors [92].

Some reports have evaluated the anti-angiogenic effect of drugs on NB cells, for example, to describe the anti-angiogenic potential of combined treatment with bortezomib and fenretinide. In this work, we used the CAM assay to analyze how a tumor graft previously generated from mouse cell lines and placed in the CAM responds to combined treatment with bortezomib and fenretinide. For this purpose, 10 embryos per group were used and treated with either PBS or 5 nmol/L bortezomib or 2.5 nmol/L fenretinide alone or their combination. The combined treatment reduced the area and density of the blood vessels to a greater extent than treatment alone. This study suggests potential therapeutic strategies for NB treatment using drug combinations [93].

Furthermore, the CAM assay was employed to evaluate the anti-angiogenic efficacy of targeted liposomal doxorubicin (TVT-DOX). Biopsy fragments of NB cell-derived xenografts, previously injected into athymic mice, were grafted onto the CAM alone (sample control) or in combination with 2 μg doxorubicin encapsulated in Caelyx (untargeted liposomes) or TVT-DOX (targeted liposomal doxorubicin; binds to the CD13 isoform expressed in the vasculature of solid tumors). In this work, the CAM assay demonstrated that TVT-DOX significantly reduced the area of vascularization compared with untreated xenografts in which DOX was administered with Caelyx [94].

In a study aiming to demonstrate the anti-angiogenic effect of vinblastine and rapamycin, both alone and in combination, the chicken CAM assay was used. Fresh biopsies obtained from patients with NB were placed in the CAM and treated with the study drugs at a concentration of 2 μmol/L for vinblastine and 5 μmol/L for rapamycin. The combined effect of both was also examined. Microscopic examination of the CAMs revealed high vascularization in the untreated tissue where the human NB biopsy was placed. However, the individual use of vinblastine and rapamycin resulted in reduced vascularization. The combination of both drugs showed significantly greater angiostatic activity. These findings are valuable and they not only confirm the anti-angiogenic activity of vinblastine and rapamycin, but also indicate the therapeutic potential of antitumor drugs when used together [95]. Additionally, the same group investigated the effects of vinblastine and rapamycin, both individually and in combination, but at lower doses (1.25 pM vinblastine and 10 pM rapamycin). They treated mouse tumor biopsy fragments inoculated with fresh cells or biopsies from patients with NB transplanted into the CAM to evaluate the effect of drug treatment on angiogenesis. After 96 h, specimens transplanted into the CAM and treated with the drug combination exhibited a significantly low number of blood vessels surrounding them compared with the untreated specimens. Moreover, the inhibitory effect on angiogenesis was notably greater with the combined drug treatment than when individually administering the drugs [96].

Ribatti et al., in 2006, reported the use of the CAM assay to evaluate the inhibitory effect of tumor-derived interferon-γ (IFN-γ) on angiogenesis. This study is noteworthy for several reasons. Prior to employing the CAM model, tumors were induced in mice with NB cell line ACN stably transfected with IFN-γ (ACN/IFN-γ) or an empty vector (ACN/neo). These tumors were subsequently fragmented to obtain fractions of 1–2 mm^3^, which were then implanted into the CAM. The authors observed a reduction in vascularization, indicating that human IFN-γ can influence NB tumor growth by inhibiting both angiogenesis and tumor proliferation. Moreover, this research suggests new possibilities for the model, as a similar approach could be applied using biopsies from human patients, allowing the preparation and administration of personalized medicine [97].

To evaluate the effect of bortezomib on in vivo angiogenesis, the CAM assay was used with the following two types of samples: fragments of neuroblastoma tumors that had been first induced in a mouse and neuroblastoma tumors from patients. Fragments of approximately 1–2 mm^3^ were implanted in the CAM of 8-day-incubated embryos and treated with either PBS (control) or 20 nM bortezomib. In addition, gelatin sponges spiked with FGF-2 (positive control) or sponges spiked with culture medium were used, both of which showed numerous radial blood vessels. Macroscopically, 96 h after both types of tumor specimens were grafted onto the CAMs, significantly fewer vessels were observed converging toward the implants in CAMs pretreated with bortezomib compared to untreated implants. These observations were confirmed via morphometric assessment of the microvessel area. The results obtained suggest that bortezomib affects tumor growth through its inhibitory effect on angiogenesis [98].

The CAM of the chicken embryo presents abundant vascularization. This characteristic allows the model to be used in angiogenesis studies. The results are reproducible, easily quantifiable, and represent a lower cost and less animal suffering compared to other angiogenesis models such as the hamster cheek pouch, the rabbit ear chamber, the rodent dorsal skin and air sac, and the iris and avascular cornea of the rodent eye [99] (Ribatti, D. and Vacca, A. (1999), Models for the study of angiogenesis in vivo. In t. J. Biol. Markers 14, 207–213.). The generation of angiogenesis-promoting signals by tumor cells causes epithelial cells to migrate within the tumor so that it is vascularized [100]. In the case of neuroblastoma, it has been observed that tumors where MYCN is overexpressed induce a greater angiogenic response compared to those in which MYCN is not amplified [88]. Therefore, this model provides the possibility of carrying out angiogenesis studies of both high and low risk neuroblastoma.

There are limitations of the CAM model for angiogenesis study in tumors because to some extent it might be difficult to distinguish between the tumor-related neo-angiogenesis and the existing embryonic neovascularization. This confusing situation may affect data interpretation. When patient-derived xenografts are placed on the CAM, penetration of the chicken vasculature into the grafts is visible 4 days after implantation and human and chicken vessels are detected. Tumor vessels are anastomosed to host vessels and then perfused. Thus, there is hybrid vessel formation between host and human capillaries in the CAM model [101].

**Table 2:** Summary of the technical characteristics in the use of the chorioallantoic membrane for the study of human NB.

Cells Used/TUMOR Type	Cell Number	Inoculation Technique	Target	Reference
**Studies focused on metastasis and antimetastatic drugs**				
UKF-NB-4 cell line	Ex ovo: 25 µL with 5 × 10^4^ cells in serum-free medium (Iscove’s modified Dulbecco’s medium or IMDM). In ovo: 25 µL with 1 × 10^6^ cells in serum-free medium (Iscove’s modified Dulbecco’s medium or IMDM).	Tumor was induced at day 10 and allowed to develop for 4 days. Tumor was induced on day 10. The tumor was allowed to develop for 6 days.	Ex ovo: To study the antiproliferative effect of CDDP (cisplatin) and Elli (ellipticin) as well as to evaluate their potential as inhibitors of cell extravasation into blood vessels. In ovo: Studying the efficiency of CDDP and Elli to inhibit metastasis of neuroblastoma (NB) cells to chick organs.	Merlos et al., 2021 [67]
SK-N-AS	1 × 10^6^ cells per microliter in serum-free medium and 2 microliters were inoculated.	Inoculation into chicken CAM was performed at day 7 cells were plated into CAM after a small laceration. Eggs were incubated until E14	Evaluating the chick embryo CAM assay for the study of metastasis.	(A. Herrmann et al., 2015; Herrmann et al., 2016) [68,69]
SK-N-AS cells	1 × 10^6^ cells resuspended in 2–10 μL of serum-free minimal essential medium.	Cells were preconditioned in 1 or 21% O_2_ for 3 days, tumors were implanted at day 7 of incubation in the chick embryo CAM. Tumors were studied at day 14 postinoculation.	To elucidate the effect of hypoxic preconditioning on the metastatic phenotype of NB cells.	(Al-Mutawa et al., 2018) [70]
+SK-N-AS MPIO-Labeled- SK-N-AS GFP-Labeled- SK-N-AS	1 × 10^6^ cells resuspended in 2–10 μL of serum-free minimal essential medium.	Cells were implanted in the CAM on day 7. Before cell inoculation the membrane was carefully lacerated. Tumors were studied at days 11 and 14.	To determine the effectiveness of Magnetic Resonance Imaging (MRI) for the evaluation of tumor development and metastasis in a chick embryo model.	(Herrmann et al., 2018) [71]
SK-N-SH	Ten million SK-N-SH cells shNEO1, shNTN4 or shSCR.	Fertilized chicken eggs were incubated and on the second day of incubation (E2), 2 mL of albumin was extracted from the egg. On the tenth day of incubation (E10), cells were plated on the developing CAM. On incubation day 17 (E17).	To provide evidence for novel roles of the NTN4/NEO1 complex in the in vivo migration, survival, and metastasis of NB cells.	(Villanueva et al., 2017) [72]
**Studies focused on chemoresistance and molecules with antitumor effect**				
UKF-NB-4 UKF-NB-4^CDDP^	1 × 10^5^ cells were inoculated.	Fertilized hen eggs were incubated for 10 days following the cell inoculation, the tumor was allowed to grow for 5 days.	To investigate the impact of upregulation of human metallothionein 3 (hMT3) in NB cells and its contribution in resistance to cisplatin treatment.	(Rodrigo et al., 2021) [79]
SK-N-AS BE(2)C	A quantity of 2 × 10^6^ GFP-labeled cells was inoculated.	Cells were inoculated on day 4 and incubated until day 14.	To investigate the potential of Palbociclib (CDK 4/6 inhibitor) and RO-3306 (CDK1 inhibitor) on NB cell differentiation, tumor progression and metastasis.	(Swadi et al., 2019) [80]
IMR32 and BE2C	2 × 10^6^ in 5 μL of DMEM or Matrigel was used.	Cells were implanted into the CAM on day 7 of embryo incubation, but before laying the cells the authors report that it is necessary to lacerate the CAM and add 5 μL of trypsin 0.05%–0.5 mM EDTA. The tumors were analyzed on day 14.	Optimize the CAM assay for the study of drugs with antitumor activity.	(Swadi et al., 2018) [81]
UKF-NB-3	5 × 10^6^ cells were resuspended in 30 μL of ECM and then implanted in CAM.	Cells were implanted on day 11 of embryo development. Eggs were incubated for another 3 days to allow formation of a distinct tumor mass. On day 14, a small silicone ring was placed around the tumor mass. On day 18, the tumors were sampled with the surrounding CAM.	To evaluate the antitumor effect of flubendazole.	(Michaelis et al., 2015) [82]
**Studies focused on angiogenesis**				
LAN-5 and GI-LI-N	3 µL suspension 6 × 10^6^ cells per ml of RPMI-1640 medium absorbedon 1 mm^3^ gelatin sponges	Tumor was induced at day 8 and processed at day 12.	Investigated 2 human NB cell lines, LAN-5 and GI-LI-N, for the capacity to induce extracellular matrix-degrading enzymes and for their angiogenic potential both in vitro and in vivo.	(Ribatti et al., 1998) [87]
HTLA-230, GI-LI-N, ACN and SH-SY5Y	Fresh in sterile RPMI-1640 (1–2 mm^3^ size). These fragments were grafted onto the CAM. Biopsy fragments (1–2 mm^3^ size) obtained from xenografts derived from four cell lines injected into nude mice.	On day 3 of incubation, a square window was opened in the eggshell after removing 2–3 mL of albumin to separate the developing CAM from the shell. On day 8, implantation was performed and on day 12, sample analysis was performed.	To evaluate the role of MYCN in the regulation of angiogenesis in NB.	(Ribatti et al., 2002) [88]
STA-NB15	2 × 10^6^ cells were transplanted. The cells were resuspended in 50 μL of collagen dissolved in cell culture medium and the mixture was solidified for 30 min at 37 °C.	On day 3 of embryonic development, 5–7 mL of egg white was removed and a 2 × 2 cm window was cut in the eggshell. On day 10, these cells were inoculated. The living embryos were incubated for another 5 days.	To understand the role of FOXO 3 as a promoter of cell migration and invasion.	(Hagenbuchner et al., 2016) [89]
Kelly	5 × 10^6^ Kelly cells were inoculated onto 15 μL Matrigel.	After 10 days, an opening was made in the eggshell and a plastic ring was placed in the CAM. Tumors were excised on day 17 of incubation.	To analyze the antitumor effect of Ang-2 upregulation in human NB.	(Klingenberg et al., 2014) [90]
UKF-NB-3	10^6^ cells were resuspended in 50 μL of medium.	The cells were inoculated onto the chicken CAM at day 8. Vessel formation was evaluated at day 12.	To analyze the effect of chemoresistance on tumor angiogenesis.	(Michaelis et al., 2009) [91]
IMR-32	Silicon rings were adsorbed with 2 μL of cell suspension of NB cells.	On day 3 of incubation a window was opened in the eggshell and 2–3 mL of albumen was removed. The angiogenic response was evaluated on day 12 of incubation after the implants.	To generate an alternative model for the study of NB angiogenesis.	(Mangieri et al., 2009) [92]
GI-LI-N	Cells were injected in the capsule of the left adrenal gland of mice. From these tumors a biopsy was taken and placed in the CAM.	On day 8 of incubation 2 mm^3^ biopsy fragments from xenograft tumors previously induced in mice, were placed onto the CAM. The CAM was analyzed at day 12.	To evaluate the antitumor effect of the administration of two combined drugs: bortezomib and fenretinide.	(Pegman et al., 2009) [93]
GI-LI-N	1 × 10^6^ cells were inoculated in the capsule of the left adrenal gland, of nude mice. A biopsy fragments taken from murine xenografts of NB.	On day 8 of incubation, biopsy fragments from xenografts derived from NB, lung, and ovarian carcinoma cells injected in athymic mice were grafted onto the CAM encapsulated in either Caelyx or in TVT-DOX. Vasculature was analyzed at day 12.	To evaluate the differences in the anti-angiogenic activity between TVT-DOX targeted liposomal doxorubicin (TVT-DOX) and Caelyx (untargeted liposomes).	(Pastorino et al., 2008) [94]
Fresh biopsy specimens from patients	1- to 2-mm^3^ fragments fresh biopsy specimens.	Biopsy fragments from patients with NB were collected under sterile conditions in RPMI 1640. This biopsy was placed inside CAM at day 8 of incubation. Analysis was performed on day 12.	To study the antitumor activity of vinblastine and rapamycin against human NB.	(Marimpietri et al., 2007) [95]
HTLA-230	1–2 mm^3^ of mouse tumor biopsy fragments Fresh biopsies from NB patients in RPMI 1640 (1 to 2 mm^3^ fragments)	On day 3, a square window was opened in the cover and 2–3 mL of albumen was removed. The window was sealed, and the eggs were returned to the incubator. On day 8, the eggs were implanted with biopsies and left until day 12.	To evaluate the synergistic effect of low doses of vinblastine (VBL) and rapamycin (RAP) on anti-angiogenesis in NB (NB).	(Marimpietri et al., 2005) [96]
NB cell line	The cell lines were inoculated into mice to generate tumors. Tumors were frozen and crushed in RPMI 1640 (1 to 2 mm^3^ fragments)	Tumor fragments were grafted into the chicken embryo CAM at day 8 of incubation. The analysis was carried out on day 12.	To evaluate the angiogenesis inhibitory effect of IFN-γ in NB-derived cells.	(Ribatti et al., 2006) [97]
HTLA-230	Grafting 1–2-mm^3^ fragments of xenograft tumors derived from cells injected into nude mice onto the CAM. Grafting fresh biopsy specimens from patients with NB in RPMI 1640 (1- to 2-mm^3^ fragments)	At day 8 of incubation, implantation of tumor fragments generated from mice was performed as tumor fragments obtained from patients were treated with PBS (control) or were treated with 20 nM bortezomib.	To analyze the effect of bortezomib on growth, apoptosis, and angiogenesis of human NB cells.	(Brignole et al., 2006) [98]

## 11. Conclusions and Perspectives

The chicken CAM model has been shown to be effective for the study of tumorigenicity, drug sensitivity, drug resistance, metastasis, and angiogenesis in tumors induced from neuroblastoma cell lines and patient-derived xenografts. A review of immense quantities of research using the chicken CAM model to study the developmental biology of NB has made it evident that the model is very flexible, economical, and easy to use. However, further investigations using diverse cell types or co-implanting other human cell types are needed to better replicate the tumor microenvironment. The CAM model offers an alternative approach for exploring the cellular and molecular biology of cancer stem cells derived from different tumors. It is crucial to continue investigating the generation of tumors in the chicken CAM, and of tumor cells from patients with NB; these potential results would allow us to study their response to treatment, achieving a study and diagnosis focused on personalized medicine. The CAM assay continues to serve as a valuable tool in the search for antitumor molecules against human NB. Moreover, it provides an opportunity to assess the potential of natural compounds and molecules with antitumor properties. In addition to chemoresistance studies, the model could be used to treat NB tumors with a radiation dose equivalent to that used in patients and to perform radiosensitivity or radioresistance studies. It is important to note that the CAM model does not replace the mouse model. Therefore, the CAM model could be used as a preliminary screening tool before scaling up to complex models, which require greater care and management.

The flexibility of this model makes it possible to use technologies including magnetic resonance imaging or ultrasound to monitor tumor growth. Furthermore, the application of omics sciences can enhance our understanding of NB biology by analyzing tumors induced in this model.

## Figures and Tables

**Table 1:** Comparison between chicken, mouse models, and cell lines for the study of tumor biology.

FEATURES	CAM	MOUSE	CELL LINE
Ease of use [38]	Yes	No	Yes
Low cost [38]	Yes	No	No
Possibility of having a large sample size [39]	Yes	No	Yes
Real-time visualization of the assays [40]	Yes	Yes	Yes
Possibility of studying metastasis [41]	Yes	Yes	No
Possibility of studying angiogenesis [42,43]	Yes	Yes	No
Maintains tumor heterogeneity [44]	Yes	Yes	No
Replicates the tumor microenvironment [36]	Yes	Yes	No
Complete accessibility to the circulatory system [36]	Yes	No	No
Requirement for animal protocol approval (country dependent) [35]	No	Yes	No
Quickly tumoral formation [44]	Yes (3 and 4 days)	No (3–4 weeks)	Are not generated
Pain perception [35]	No (until day14)	Yes	No
Direct visibility of the tumor [44]	Yes	No	No
Availability of reagents (antibodies, primers, etc.) [36]	No	Yes	Yes
Immunodeficiency [45]	Yes	Yes	Yes
Genetic closeness to humans [46,47,48]	No	Yes	Yes
